# Grainyhead‐like 2 (GRHL2) regulates epithelial plasticity in pancreatic cancer progression

**DOI:** 10.1002/cam4.1212

**Published:** 2017-09-29

**Authors:** Hitoe Nishino, Shigetsugu Takano, Hideyuki Yoshitomi, Kensuke Suzuki, Shingo Kagawa, Reiri Shimazaki, Hiroaki Shimizu, Katsunori Furukawa, Masaru Miyazaki, Masayuki Ohtsuka

**Affiliations:** ^1^ Department of General Surgery Graduate School of Medicine Chiba University Chiba Japan

**Keywords:** Cancer stem cell, epithelial plasticity, GRHL2, mesenchymal‐epithelial transition (MET), pancreatic cancer

## Abstract

The epithelial‐mesenchymal transition (EMT) and mesenchymal‐epithelial transition (MET) contribute to cancer metastasis of pancreatic ductal adenocarcinoma (PDAC). We explored the role of grainyhead‐like 2 (GRHL2), a suppressor of EMT, in the progression of PDAC. Expressions of GRHL2 were assessed using surgically resected PDAC tissues by immunohistochemistry analysis, and in vitro using human and mouse PDAC cells. Effects on epithelial plasticity and stemness of GRHL2 were examined in vitro using liver metastatic PDAC cells (CFPAC‐1) with GRHL2 knockdown by specific siRNAs. GRHL2 has a significantly positive correlation with E‐cadherin and CD133 in 155 resected human primary PDAC tissues. GRHL2 is highly expressed in liver metastatic cells than in primary invasive cells of both human and mouse PDAC, accompanied by a positive correlation with E‐cadherin expression. GRHL2 knockdown CFPAC‐1 cells demonstrated morphological changes into mesenchymal appearances and reduced proliferation through EMT. Notably, knockdown studies followed by flow cytometry analysis for a subpopulation of CD133+ showed that GRHL2 facilitates CFPAC‐1 cells to maintain stem‐like characters including self‐renewal capacity and anoikis resistance. GRHL2 regulates epithelial plasticity along with stemness in PDAC, both of which are crucial for metastasis, implicating the possibility of GRHL2 as a therapeutic target for PDAC liver metastasis.

## Introduction

Pancreatic ductal adenocarcinoma (PDAC) is the fourth leading cause of cancer‐related deaths 1. Despite the advances in surgery and chemotherapy for PDAC, 5‐year overall survival even for patients with surgically resected PDAC remains about 20–25%, accompanied by high rate of recurrent systemic disease 2. This trait of easily becoming an extensive metastatic disease can be partially explained with the recent finding that hematogenous spread of circulating tumor cells from the pancreas appears to be an early event in PDAC progression 3. Specifically, cells within histological “preinvasive” pancreatic intraepithelial neoplasia (PanIN) can circulate into the bloodstream, and seed the liver in genetically engineered mouse models.

Epithelial‐mesenchymal transition (EMT) plays an important role in tumor progression and metastasis in diverse cancers including PDAC 4. A variety of intrinsic and extrinsic factors initiate and propagate the signaling during EMT or the reverse process, the mesenchymal‐epithelial transition (MET), leading to alterations in the expression of several transcription factors 5. However, the molecular mechanisms that regulate EMT and MET in cancer progression remain unclear.

Grainyhead‐like 2 (GRHL2) is a member of grainyhead‐like transcription family, which plays a fundamental role in epidermal integrity, embryonic neural tube closure, and wound healing processes 6. Emerging evidence has revealed that GRHL2 is a novel proto‐oncogene that regulates epithelial plasticity by suppressing EMT in several tumor types 7, 8. Recent studies focused on demonstrating its crucial role in regulating EMT and MET in cancer cells 9, 10. GRHL2 inhibits TGF‐*β* mediated activation of Smad2/3, which leads to the loss of ZEB1 expression and accompanying upregulation of miR‐200 expression 11. It is also revealed that GRHL2 inhibits transactivation of the ZEB1 promoter mediated by the homeodomain proteins Six1, LBX1, and HoxA5. ZEB1 reciprocally repressed GRHL2 expression through a direct interaction with the GRHL2 promoter 7. Furthermore, Yang et al. have shown the expressional association and clinical relevance of six genes (*CDH2, FN1, CITED2, CTNNB1,* and *CTNNA3*) identified as *GRHL2*‐related genes together with *GRHL2* for breast cancer metastasis 12. In contrast, GRHL2 is considered as a tumor suppressor in gastric cancer, cervical cancer, clear cell renal cell carcinoma, and sarcoma 13, 14. Thus, the functional roles and clinical impact of GRHL2 vary with cancer type, and its expression and effect in pancreatic carcinogenesis has not yet been investigated.

Herein, we explored and defined novel functional roles for GRHL2 in the regulation of EMT and MET during cancer progression in PDAC cells. GRHL2 suppresses EMT and drives invasive PDAC cells to alter epithelial phenotype with self‐renewal capacity to induce metastatic colonization. This study contributes to a better understanding of the functional roles of GRHL2 in PDAC, which might prove to be a novel therapeutic target for PDAC.

## Materials and Methods

### Human and murine pancreatic cell lines

Human pancreatic duct epithelial (HPDE) cell line was provided by Dr. Rustgi (University of Pennsylvania) 15. Human PDAC cell lines (PANC‐1, BxPC‐3, MIA PaCa‐2, AsPC‐1, Hs 766T, CFPAC‐1) 16 were procured from the American Type Culture Collection (Manassas, VA, USA). Murine cells of PanIN (KC), PDAC (KPC1 and KPC2), and the paired liver metastases (KPC1Liv and KPC2Liv) were provided by Dr. Hingorani (University of Washington). Briefly, KC cells were isolated from a mouse at the PanIN stage (*LSL‐Kras* ^*G12D*/+^; *Pdx1‐cre*). Both KPC1 and KPC2 cell lines (*LSL‐Kras* ^*G12D*/+^; *p53* ^*R172H*/+^; *Pdx1‐cre*) were established from primary PDAC in KPC mice 17, whereas the KPC1Liv and KPC2Liv cell lines (*LSL‐Kras* ^*G12D*/+^; *p53* ^*R172H*/+^; *Pdx1‐cre*) were isolated from paired liver metastases arising in KPC1 and KPC2 mice.

### Cell culture

PANC‐1, MIA PaCa‐2, Hs 766T, and all the mouse pancreatic cells were cultured in Dulbecco's modified Eagle medium (DMEM: Sigma‐Aldrich, St Louis, MO, USA) with 10% fetal bovine serum (FBS), CFPAC‐1 in Iscove's modified Dulbecco's medium (IMDM: Thermo Fisher Scientific, Waltham, MA, USA) with 10% FBS, BxPC‐3, and AsPC‐1 cells in RPMI‐1640 medium (Thermo Fisher Scientific) with 10% FBS, and HPDE cells in keratinocyte serum‐free medium supplemented with bovine pituitary extract, epidermal growth factor (KSFM: Thermo Fisher Scientific), and antibiotics (1% penicillin and streptomycin).

### Quantitative RT‐PCR

Quantitative RT‐PCR was performed with SYBR Green in a real‐time PCR system (Applied Biosystems, Foster City, CA, USA) for total RNA purified with RNeasy Mini Kit (Qiagen Inc., Valencia, CA, USA) according to the manufacturer's instructions. GRHL2 primers are as follows: GRHL2‐F, 5′‐GGGCATAGGACTCCAGAGTAGGAA‐3′; GRHL2‐R, 5′‐TAGGGCAGGACTGGCAAACA‐3′ (TAKARA BIO INC., Kusatsu, Shiga, Japan). The comparative *C*_T_ method was used to determine relative gene expression levels for each target gene.

### Western blot analysis

Western blot was performed via established protocols for total cellular proteins extracted with RIPA Buffer (Sigma‐Aldrich). Primary antibodies used were: human GRHL2 (HPA004820: Sigma‐Aldrich, 1:500), mouse GRHL2 (sc‐87143: Santa Cruz Biotechnology, Santa Cruz, CA, USA; 1:100), Vimentin (sc‐6260: Santa Cruz Biotechnology; 1:500), E‐cadherin (sc‐7870: Santa Cruz Biotechnology; 1:1000), *β*‐actin (Cell Signaling Technology, Danvers, Massachusetts, USA; 1:2000). Band intensities from the western blot were quantified by densitometry analysis and normalized to *β*‐actin using the Image J software.

### RNA interference and reagents

We chose CFPAC‐1 cell line for GRHL2 knockdown experiments as it has strong GRHL2 expression compared to other human PDAC cell lines. Double‐stranded small interfering RNAs used to knockdown GRHL2 were as follows: siRNA1:Hs_GRHL2_2 Cat#SI04150090, siRNA2:Hs_GRHL2_3 Cat#SI04275271 (Qiagen Inc.). Control cells were treated with negative control siRNA (AllStars negative control siRNA; Qiagen Inc.). Cells precultured for 24 h were transfected with these siRNAs (10 nmol/L final concentration), and knockdown efficiency was assessed by western blot 72 h posttransfection.

### Three‐dimensional cell culture

Three‐dimensional (3D) organotypic pancreatic ductal cell culture was performed as described previously 18. Briefly, cells suspended in collagen I solution were seeded onto collagen‐coated four‐well chamber slides (Thermo Fischer Scientific, Rochester, NY, USA). After 10 days, cells were imaged using the Axiovert 25 inverted microscope (Carl Zeiss, Oberkochen, Germany). The structures were classified into three types—spheroid cysts, irregular cysts, and spindle‐shaped cells—and their numbers were determined.

### Cell proliferation assay

Cell Counting Kit‐8 (CCK‐8: Dojindo Laboratories, Kumamoto, Japan) was used to assess cell proliferation following the manufacturer's protocol. Cells were seeded onto 96‐well plates (2000 cells/well) and cultured for 24, 48, 72, 96, and 120 h. The absorbance at 450 nm was measured with a microplate reader.

### Cell cytotoxicity assay

Cells (500 cells/well) were seeded onto 96‐well plates, precultured for 24 h and treated with the indicated concentration (200 ng/mL) of gemcitabine hydrochloride (Sigma‐Aldrich) for 4 days. We quantified the number of viable cells daily with Cell Counting Kit‐8 (Dojindo Laboratories).

### Pancreatosphere formation assay

Cells (10 cells/well) were seeded onto 96‐well ultralow attachment plates (Corning, New York, NY, USA). After 7 days in sphere medium 19, we counted the number of sphere cells that were defined as cell clusters with over 50 *μ*m diameters on day 7. The sphere formation rate was assessed as the percent increase in the number of spheres on day 7 with respect to the number of spheres observed on day 1.

### Anoikis assay

Anoikis assay was performed as described previously 20. In brief, cells at low density (30,000 cells/mL) were continuously rotated on tube rotator **(**HB‐1000 Hybridizer Hybridization Oven; Ultra‐Violet Products, Upland, CA, USA) for 24 h. Then, 3000 cells/well suspended in medium with 0.3% agar were seeded on to the 24‐well culture plates coated with bottom layer (medium with 1% agar), and were cultured for 14 days. Tumor colonies were visualized after staining with Giemsa Stain Solution (WAKO, Osaka, Japan, dilution 1:20) to reveal the number and size of the colonies formed.

### Flow cytometry analysis by fluorescence‐activated cell sorting

Two million cells were suspended in 100 *μ*L PBS and incubated with APC‐labeled anti‐human CD133 antibody (Beckton‐Dickinson, Flanklin Lakes, NJ, USA), anti‐human CD326 (EpCAM) antibody (Miltenyi Biotec, K.K., Tokyo, Japan) for 60 min on ice in the dark. After washing with PBS, cells were resuspended in 1 mL PBS and acquired on CANTO II system (Beckton‐Dickinson). Data were analyzed using FlowJo v10.1r5 software (FlowJo, LLC, Ashland, OR, USA).

### Patients and human tissue samples

PDAC tissues were obtained from 155 consecutive patients who underwent surgical resection in the Department of General Surgery, Chiba University Hospital, Japan, from February 2006 to November 2011. All patients were diagnosed with primary and liver metastatic PDAC histologically. All small metastatic liver tumors were resected from the surface of liver. The Ethics Committees of our institute approved the protocol of this study and written informed consent was obtained from each patient before surgery.

### Immunohistochemistry

Paraffin‐embedded tissue specimens of PDAC were evaluated for GRHL2, E‐cadherin, Vimentin, and CD133 expression. Immunohistochemistry (IHC) was performed according to the manufacturer's instructions. The fixed tissues were incubated overnight at 4°C with primary antibodies for GRHL2 (Sigma‐Aldrich, 1:1000), E‐cadherin (Santa Cruz Biotechnology, 1:1000), Vimentin (Santa Cruz Biotechnology, 1:500), or CD133 (AC133: Miltenyi Biotec, Bergisch Gladbach, Germany; 1:100). EnVision^™^ kit (Dako, Glostrup, Denmark) and DAB were used for visualization. The staining intensities of GRHL2, E‐cadherin, Vimentin, and CD133 were evaluated independently by two investigators (HN, ST) with a pathologist and scored as follows: low expression: 0–50% tumor cells stained positive; high expression: more than 50% tumor cells stained positive. The staining intensity of normal pancreatic ductal cells was used as an internal positive control.

### Statistical analysis

Accumulative rates were calculated by the Kaplan–Meier method and the significance of difference in survival rate was analyzed by the log‐rank test. Data are expressed as mean ± standard deviation (SD) or the standard error of the mean (SEM). Statistical significance of the results were determined by Student's *t*‐test, Welch's *t*‐test, chi‐square test, or Fisher's exact test. *P *<* *0.05 was considered significant in all analyses. All statistical calculations were performed using the Statview‐J5.0 software package (SAS Institute Inc., Cary, North Carolina, USA).

## Results

### GRHL2 expression positively correlates with E‐cadherin and CD133 in primary PDAC and is highly expressed in liver metastatic PDAC human tissues

**Figure 1:**
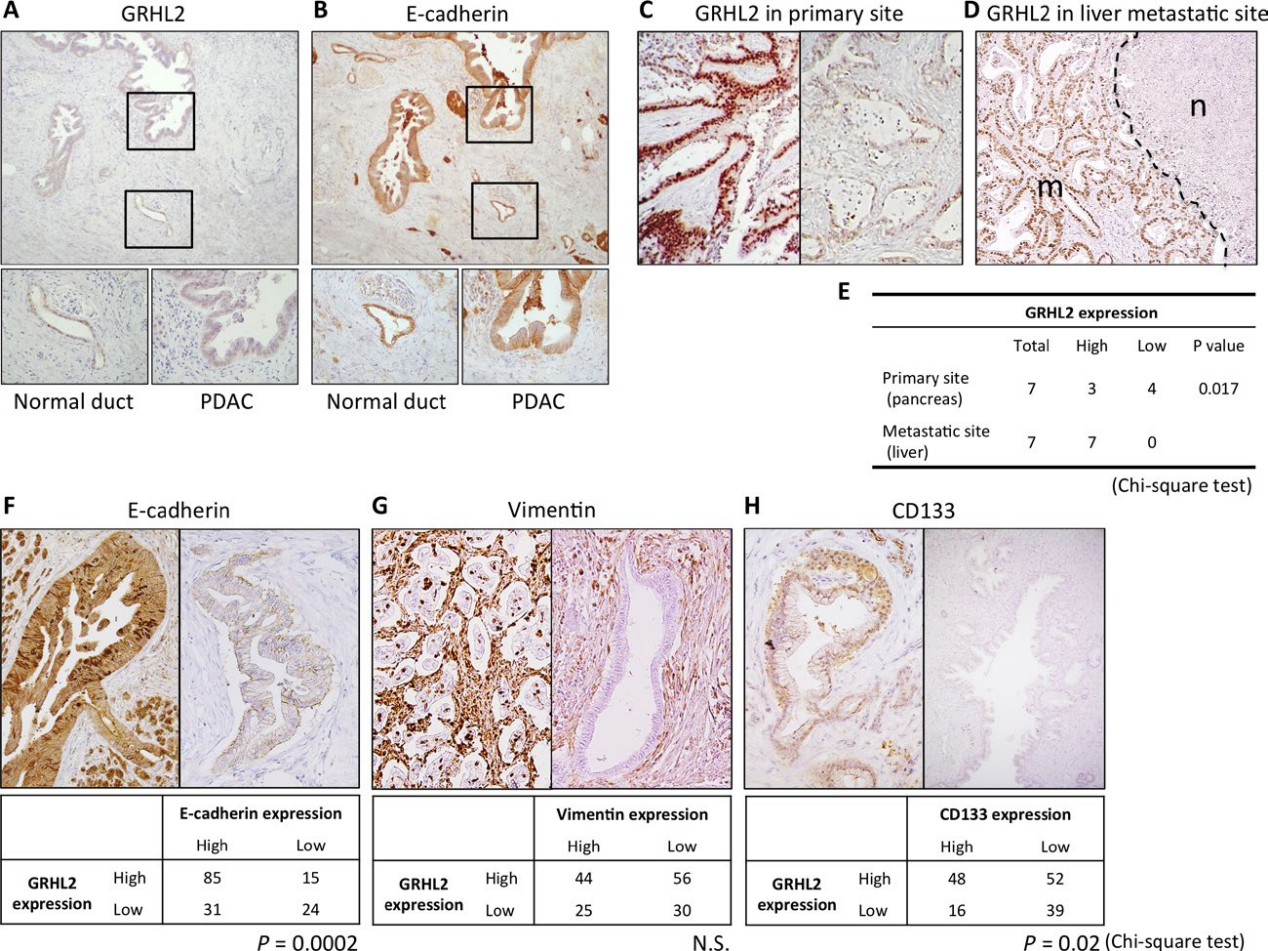
IHC analyses and the correlation between GRHL2 and EMT/CSC markers in human PDAC samples. (A–B) GRHL2 (A) and E‐cadherin (B) expression in normal pancreatic duct and invasive pancreatic ductal cells. (upper panel: 100×, lower panel: 400×) (C) The nuclei staining of GRHL2 in primary PDAC tissue was categorized into high expression (left panel) or low expression (right panel) based on the intensity criterion (refer Materials and Methods) (*n* = 155). (D) IHC staining of GRHL2 in liver metastatic tumor of PDAC (n: adjacent normal liver lesion, m: liver metastasis lesion). (E) Comparison of IHC staining intensity for GRHL2 between primary and metastatic PDAC in pair‐matched samples (*n* = 7, *P* = 0.017). (F–H) The staining intensity of E‐cadherin (F), Vimentin (G), and CD133 (H) in primary PDAC tissue was evaluated and categorized into two groups (High expression: upper left panel, Low expression: upper right panel). Comparison of IHC staining intensity for GRHL2 and E‐cadherin (F), Vimentin (G), and CD133 (H) in primary PDAC tissues (lower table) (*n* = 155). *P* values are indicated below the respective tables. N.S.: not significant. Original magnification: 400×.

At first, we analyzed GRHL2 expression in human primary PDAC tissues by IHC staining. GRHL2, predominantly localized in the nucleus of cells, is strongly expressed in normal pancreatic duct compared to invasive pancreatic ductal cells (Fig. 1A). The expression pattern of GRHL2 is similar to that of E‐cadherin localized in the membrane and cytoplasm of cells (Fig. 1B). Among 155 cases, 100 cases (64.5%) showed high GRHL2 expression, while 55 cases (35.5%) showed low GRHL2 expression based on the staining score (refer Materials and Methods) (Fig. 1C). The patient characteristics of GRHL2 high/low groups are summarized in Table 1. There were no significant differences between the backgrounds of these two groups in primary PDAC tissues. We next assessed GRHL2 expression in primary tumors and liver metastases of PDAC patients. GRHL2 is highly expressed in liver metastases of PDAC (Fig. 1D) (7/7: 100%, high group), and notably, GRHL2 expression in liver metastasis is significantly higher than in paired primary tumor obtained from the same PDAC patient (*n *=* *7) (*P *=* *0.017; chi‐square test; Fig. 1E). Considering the biological functional roles for GRHL2 in the EMT/MET plasticity, we performed IHC staining for epithelial/mesenchymal markers, E‐cadherin and Vimentin to investigate the correlation between GRHL2 and, these markers in primary PDAC tissues. Interestingly, GRHL2 and E‐cadherin expression had a strong positive correlation (*P *=* *0.0002; Fig. 1F), whereas there was no correlation between GRHL2 and Vimentin expression in primary PDAC tissues (Fig. 1G). These observations implicated that GRHL2 is associated with epithelial features in human PDAC.

**Table 1:** Correlation of GRHL2 expression with clinicopathological findings in PDAC

Parameters	GRHL2 expression			
	Total	High (*n *=* *100)	Low (*n *=* *55)	*P*‐value
Age (year)	65.9 ± 9.1	65.5 ± 9.4	66.6 ± 8.4	0.76
Gender (M/F)	97/58	64/36	33/22	0.62
Histology (well, mod/poor)	134/21	89/11	45/10	0.21
pT stage (T1, T2/T3, T4)	7/148	4/96	3/52	0.67
pN stage (0/1)	41/114	27/73	14/41	0.83
UICC‐stage (IB, IIA, IIB/III, IV)	147/8	96/4	51/4	0.38

p, pathological findings; T, primary tumor, N, regional lymph nodes (chi‐square test).

We also analyzed the correlation of GRHL2 and CD133 expression in primary PDAC tissues. CD133 showed predominant membrane localization in primary PDAC cells (Fig. 1H). High CD133 expression significantly correlated with high GRHL2 expression in primary PDAC tissues (*P *=* *0.02). These results also implicated that GRHL2 expression might be linked to the cancer stem cell (CSC)‐like property in human PDAC tissues.

### GRHL2 is highly expressed in metastatic pancreatic cells characterizing epithelial property

**Figure 2:**
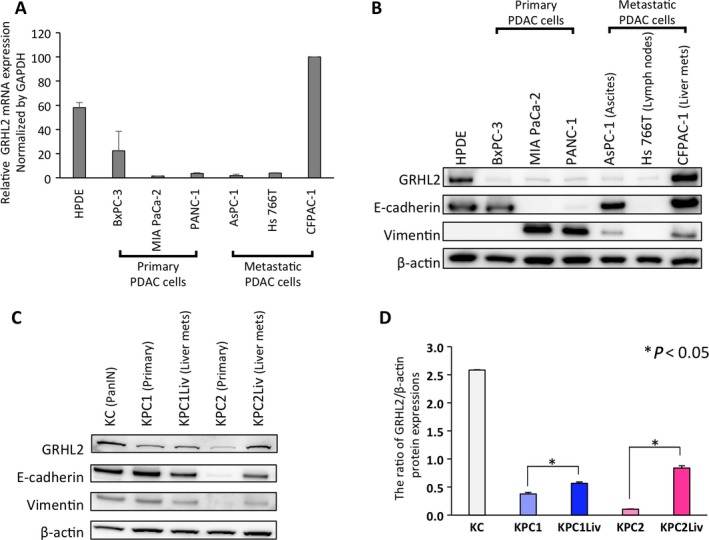
Differential expression of GRHL2 in different human/mouse pancreatic ductal cell lines. (A–B) GRHL2 expression in various human pancreatic cell lines derived from normal pancreas (HPDE), primary PDAC (BxPC‐3, MIA PaCa‐2, PANC‐1), metastatic ascites (AsPC‐1), lymph node metastasis (Hs766T) and PDAC liver metastases (CFPAC‐1) was determined by qRT‐PCR analysis (A) or western blot analysis (B). (C) GRHL2 protein expression levels in established mouse cell lines derived from pancreatic intraepithelial neoplasia (PanIN) cells (KC), PDAC in primary site (KPC1, KPC2) and its metastatic site in liver (KPC1Liv, KPC2Liv) were analyzed by western blot. (D) Comparative analysis of GRHL2 expression among five cell lines. The band intensities were normalized to that of *β*‐actin. Experiments were performed in triplicate. Error bars represent the standard deviation (SD).

Next, we investigated GRHL2 expression in human pancreatic ductal cells. Increased GRHL2 mRNA and protein expression was found in human pancreatic ductal epithelial (HPDE) cells as well as CFPAC‐1, liver metastatic PDAC cells, whereas primary invasive PDAC cells displayed decreased expression (Fig. 2A and B). We also assessed the correlation of protein expression between GRHL2 and representative epithelial/mesenchymal markers, E‐cadherin and Vimentin, in these cell lines. E‐cadherin is highly expressed in both HPDE and CFPAC‐1 cells with high GRHL2 expression, whereas, Vimentin expression is high in primary PDAC cells (Fig. 2B). Next, we examined GRHL2 protein expression in murine pancreatic cells isolated from pancreas of KC mice or pancreas/liver metastasis of KPC mice, genetically engineered mouse models. Consistent with the results from human cell lines, GRHL2 showed high expression in KC precancerous cells and liver metastatic KPC cells characterizing epithelial phenotype compared to primary KPC cells exhibiting mesenchymal phenotype (Fig. 2C). Interestingly, E‐cadherin is synchronously expressed with GRHL2 in these murine cells. Additionally, liver metastatic cells compared to pair‐matched primary tumor cells show a significant increase in relative GRHL2 expression (Fig. 2D). Taken together, these data supported our findings of human PDAC tissues to confirm the association between GRHL2 and cells characterizing epithelial phenotype in pancreatic cells.

### GRHL2 regulates epithelial morphology and cell proliferation in PDAC cells

**Figure 3:**
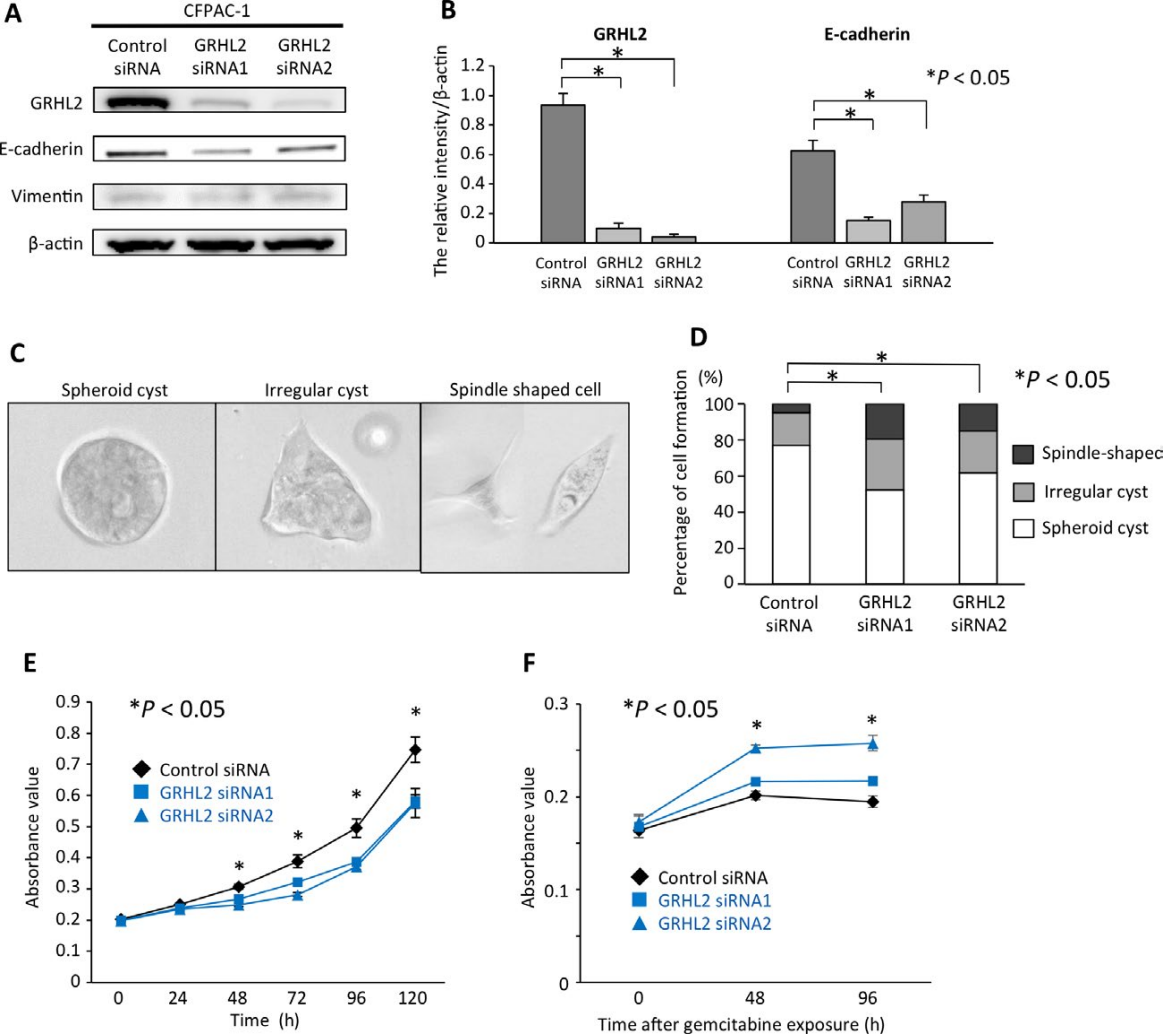
Morphological and functional change into mesenchymal phenotype upon GRHL2 knockdown in CFPAC‐1 cells. (A) GRHL2, E‐cadherin, and Vimentin expression by western blot in CFPAC‐1 cells treated with GRHL2 specific siRNAs. (B) Comparative analysis of GRHL2 expression between cells treated with control and GRHL2 siRNAs. The band intensities were normalized to that of *β*‐actin (*P <* *0.05). (C) Representative pictures of CFPAC‐1 cells in three‐dimensional culture. Spheroid cyst (left panel), irregular cysts (middle panel), and spindle shaped cells (right panel). (D) The percentages of spheroid cyst, irregular cyst, or spindle shaped cell formation (*P < 0.05). (E) Cell proliferation assay. The relative number of CFPAC‐1 cells treated with GRHL2 siRNA1 or GRHL2 siRNA2 was compared to the control cells at indicated hours (*P < 0.05). (F) Cell cytotoxicity assays. The relative number of CFPAC‐1 cells treated with GRHL2 siRNA1 or GRHL2 siRNA2 was compared to the control cells at 48 and 96 h after gemcitabine exposure (*P < 0.05). Error bars represent SD.

To explore the functional roles of GRHL2 in PDAC cells, endogenous GRHL2 expression was suppressed by two specific siRNAs for GRHL2 (GRHL2 siRNA1, GRHL2 siRNA2). Knockdown of GRHL2 resulted in significant downregulation of E‐cadherin in CFPAC‐1 cells (Fig. 3A and B). Using an established three‐dimensional (3D) organotypic culture system, we investigated whether GRHL2 influences the morphology of PDAC cells. CFPAC‐1 control cells expressing GRHL2 formed well‐organized spheroid cysts, whereas GRHL2 knockdown cells demonstrated altered morphology and larger population of mesenchymal spindle shaped cells compared to control cells (Fig. 3C and D). These results suggest that GRHL2 plays a functional role in maintaining the epithelial phenotype in PDAC cells.

We also examined whether GRHL2 affects PDAC cell proliferation. GRHL2 knockdown resulted in significantly reduced proliferation as compared to CFPAC‐1 control cells (*P *<* *0.05) (Fig. 3E). Recent experimental evidences have proposed that EMT is associated with chemo resistance 4. Toward understanding, if GRHL2 is crucially involved in the maintenance of epithelial phenotype in PDAC cells, we hypothesized that GRHL2 knockdown induces mesenchymal phenotype and renders them more resistant to chemotherapy‐induced cytotoxicity through EMT process. To analyze this hypothesis, we performed cytotoxicity assays with gemcitabine, which is a widely used chemotherapeutic reagent for PDAC. Upon gemcitabine treatment, GRHL2 knockdown CFPAC‐1 cells exhibited enhanced resistance against cytotoxicity as compared to CFPAC‐1 cells (Fig. 3F). This result demonstrated that GRHL2 knockdown facilitates EMT and strengthens their chemo resistance. Taken together, these results indicate that GRHL2 induces cell proliferation and sensitizes PDAC cells to chemotherapy‐induced cytotoxicity along with epithelial phenotype.

### GRHL2 maintains stem‐like characteristics in liver metastatic PDAC cells

**Figure 4:**
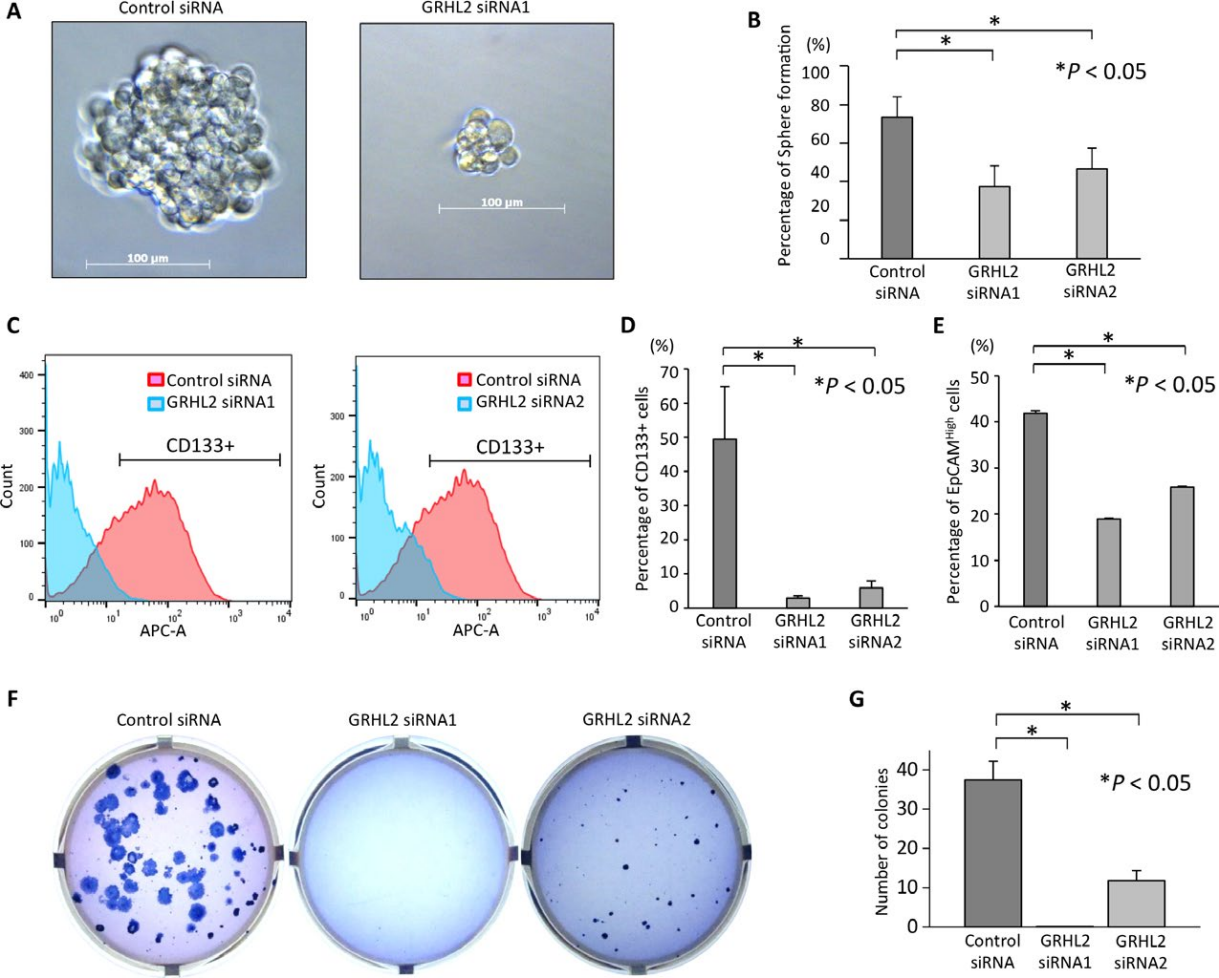
GRHL2 maintains the property of stemness in liver metastatic PDAC cells. (A) Representative sphere formation in CFPAC‐1 cells treated with control siRNA (left panel) or GRHL2 knockdown CFPAC‐1 cells (right panel). (B) The sphere formation rate was evaluated among these three samples treated with siRNAs (*P <* *0.05). (C) Flow cytometry analysis for CD133 expression in CFPAC‐1 cells treated with siRNAs. (D) The percentage of subpopulation of CD133+ cells in CFPAC‐1 cells treated with control, or two GRHL2 siRNAs. (control: 49.5%, siRNA1: 2.9%, siRNA2: 5.9%, *P <* *0.05). Error bars represent SEM. (E) The percentage of subpopulation of EpCAM^High^ cells in CFPAC‐1 cells treated with control, or two GRHL2 siRNAs. (control: 41.8%, siRNA1: 18.9%, siRNA2: 25.8%, *P <* *0.05). Error bars represent SEM. (F) Representative figures showing colony formation in CFPAC‐1 cells treated with control, or GRHL2 siRNAs 14 days after cell seeding by anoikis assay. (G) Number of colonies among CFPAC‐1 cells treated with siRNAs (*P <* *0.05). Error bars represent SD.

In order to colonize at a distant organ, self‐renewal properties are required for the disseminating cancer cells. We performed pancreatosphere formation assay to investigate whether GRHL2 confers the property of putative cancer stem cell (CSC) in CFPAC‐1 cells. In pancreatosphere formation assays, GRHL2 knockdown CFPAC‐1 cells demonstrated significantly reduced number of spheres compared to control CFPAC‐1 cells (Fig. 4A and B). These results indicate that GRHL2 maintains the self‐renewal capacity in liver metastatic PDAC cells.

We next examined whether GRHL2 expression correlates with that of CD133, a representative CSC marker for PDAC 21. Flow cytometry analyses showed increased CD133+ cells in CFPAC‐1, liver metastatic PDAC cells compared to PANC‐1, primary PDAC cells (data not shown). Notably, the percentage of CD133+ cells dramatically reduced in GRHL2 knockdown CFPAC‐1 cells compared to control cells (*P *<* *0.05; Fig. 4C and D). Additionally, the percentage of EpCAM^High^, another CSC marker for PDAC significantly decreased in GRHL2 knockdown CFPAC‐1 cells compared to control cells (*P *<* *0.05; Fig. 4E). These results suggest that GRHL2 might play a crucial role of stem‐like property of liver metastatic PDAC cells.

### GRHL2 is necessary for anoikis resistance in metastatic PDAC cells

Lastly, we sought to evaluate whether GRHL2 affects anoikis resistance of PDAC cells. In order to form metastatic colonization, disseminating tumor cells that enter into the bloodstream need to survive from apoptosis termed anoikis that results from losing the attachment of those cells to the extracellular matrix 22. The anoikis resistance ability is a major characteristic of CSC that promotes metastasis 20, 23. GRHL2 positive control cells showed increased colony formation and the colonies were larger compared to GRHL2 knockdown cells (Fig. 4F and G), suggesting that GRHL2 contributes to the maintenance of anoikis resistance in CFPAC‐1 cells in vitro. Taken together, these results imply that GRHL2 enhances the CSC‐like characters in liver metastatic PDAC cells with the ability to form spheres and anoikis resistance, which facilitates these cells to survive and colonize metastasis at distant organs.

## Discussion

**Figure 5:**
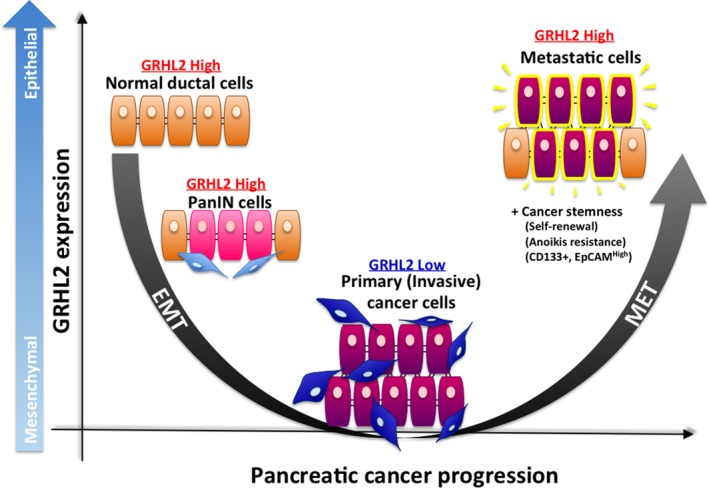
The schema of relationship between GRHL2 expression and PDAC progression. Pancreatic ductal cells and PanIN cells show high expression of GRHL2 and epithelial phenotype. On the contrary, PDAC cells at primary site show low expression of GRHL2, which is accompanied by invasive characteristics as they change into mesenchymal cells through EMT. Liver metastatic PDAC cells show high expression of E‐cadherin, which is one of the representative epithelial markers, and high reexpression of GRHL2. Collectively, GRHL2 plays an important role in regulating epithelial plasticity in PDAC progression and metastasis.

In this study, we demonstrated for the first time that GRHL2 has a functional role in the regulation of epithelial plasticity of PDAC cells (Fig. 5). IHC analyses revealed that GRHL2 expression showed strong positive correlation with both E‐cadherin and CD133 in clinical samples. Our in vitro data also suggested that GRHL2 facilitates invasive PDAC cells to undergo MET in order to prevent anoikis.

EMT and MET are thought to be vital cellular characteristics in the invasion‐metastasis cascade of cancers 24. EMT and MET enable cancer cells to survive and induce metastatic colonization post dissemination and extravasation into the parenchyma of distant organs. The metastatic tumors almost invariably display epithelial features, such as well‐organized adherens junctions, suggesting that tumor cells disseminating from the primary site revert to an epithelial phenotype through MET as they form macroscopic metastases in secondary sites 25. Recent studies revealed the significance of EMT in PDAC carcinogenesis 3; however, the involvement of MET in PDAC metastasis is poorly understood. In this study, we hypothesized that GRHL2 could be a potential therapeutic target regulating epithelial plasticity and CSCs‐like property of PDAC. We examined the correlation between GRHL2 and Vimentin, a representative mesenchymal marker for human PDAC tissues. Unexpectedly, there was no inverse correlation between GRHL2 and Vimentin expression; however, we confirmed that GRHL2 expression strongly correlates with E‐cadherin or CD133 expression in PDAC tissues. The fact that CD133 is expressed specifically in pancreatic ductal epithelium supports our results 26.

In this study, we demonstrated that GRHL2 is upregulated in human pancreatic ductal cells and mouse PanIN cells compared to primary invasive PDAC cells. Notably, both GRHL2 and E‐cadherin in human and mouse PDAC cell lines with liver metastases appeared to be significantly upregulated, which suggests the observation that reestablishing epithelial integrity is required for disseminating cancer cells to form overt metastasis 10, 27. We confirmed that GRHL2 knockdown promotes the morphological change from epithelial to mesenchymal phenotype in three‐dimensional culture. It is also speculated that GRHL2 knockdown accelerates cells to acquire mesenchymal traits in precancerous PanIN cells. This finding suggests that GRHL2 might be required to determine epithelial phenotype of pancreatic ductal cells during PDAC progression.

Cell proliferation is an epithelial characteristic crucial for macrometastasis. Previous studies demonstrated that GRHL2 knockdown resulted in a significant reduction in cell proliferation in various cancer cells 28. Consistent with this, we demonstrated that GRHL2 downregulation decreased the PDAC cell proliferation. Collectively, GRHL2 appears to be the essential transcription factor that determines the epithelial plasticity of PDAC and contributes to maintain epithelial functional traits. The role of EMT in metastasis is a longstanding source of debate. Recent studies support a novel proposal that EMT is not always essential for metastasis 29. However, EMT cells significantly contribute to recurrent lung metastasis formation after chemotherapy 30, and suppression of EMT leads to an enhanced sensitivity to gemcitabine treatment 31. Our study demonstrated that knockdown of GRHL2 induces cytotoxicity resistance as well as EMT in PDAC cells. The drug resistance appears to be more influenced by mesenchymal phenotypic change induced by GRHL2 knockdown in liver metastatic PDAC cells.

In this study, we also demonstrated that GRHL2 knockdown decreases the subpopulation of CD133+ cells or EpCAM^High^ cells in metastatic PDAC cells, supporting the observation that GRHL2 regulates liver metastatic PDAC cells to maintain stem cell‐like properties. Increasing evidence indicates that the tumor cells that initiate metastatic outgrowth are CSCs or, at least possess several attributes of these cells 32. Some studies indicate the effects of CSC on metastasis; a distinct subpopulation of CD133+ CXCR4+ CSCs was identified that determines the metastatic phenotype of the individual tumor 21. It has been demonstrated that EMT‐inducing transcription factors confer mesenchymal as well as stem cell properties 26, 33, whereas results of the counterpart that EMT can suppress CSC‐like characteristics including self‐renewal capacity were also shown in previous studies 34. Understanding this discrepancy, we investigated self‐renewal capacity and anoikis resistance, which are two stem cell‐like properties required to survive and outgrow in the hostile environment of a distant organ in vitro. Notably, both self‐renewal capacity and anoikis resistance were GRHL2‐dependent in liver metastatic PDAC cells with epithelial phenotype. Collectively, our results suggest that GRHL2 plays a crucial role in regulating epithelial plasticity with CSC‐like properties to form overt liver metastatic colonies.

In conclusion, our study demonstrated that MET and CSC‐like properties might be coupled by GRHL2, which is associated with epithelial characteristics and enhanced stemness of PDAC. These findings provide an insight into plasticity regulation during PDAC progression. Further studies are warranted to examine the functional relationship between GRHL2 and epithelial phenotype in in vivo assays such as orthotopic transplantation and metastatic models, and determine whether GRHL2 could be a promising therapeutic target for PDAC.

## Conflict of Interest

The authors declare no conflict of interest associated with this manuscript.
